# Optimization of Aging Temperature and Heat-Treatment Pathways in Additively Manufactured 17-4PH Stainless Steel

**DOI:** 10.3390/ma16247557

**Published:** 2023-12-08

**Authors:** Hobyung Chae, Sangyeob Lim, Taeho Lee, Eunjoo Shin, Joowon Suh, Suk Hoon Kang, Soo Yeol Lee

**Affiliations:** 1 Neutron Science Division, Korea Atomic Energy Research Institute, Daejeon 34057, Republic of Korea; hob2021@kaeri.re.kr (H.C.); it-sej@kaeri.re.kr (E.S.); 2 Nuclear Materials Division, Korea Atomic Energy Research Institute, Daejeon 34057, Republic of Korea; sylim@kaeri.re.kr (S.L.); jwsuh97@kaeri.re.kr (J.S.); shkang77@kaeri.re.kr (S.H.K.); 3 Department of Materials Science and Engineering, Chungnam National University, Daejeon 34134, Republic of Korea; dlxogh1021@o.cnu.ac.kr

**Keywords:** additive manufacturing, 17-4PH stainless steel, heat-treatment

## Abstract

This study investigates the tensile behaviors of additively manufactured (AM) 17-4PH stainless steels heat-treated within various temperature ranges from 400 °C to 700 °C in order to identify the effective aging temperature. Despite an aging treatment of 400–460 °C increasing the retained austenite content, an enhancement of the tensile properties was achieved without a strength-ductility trade-off owing to precipitation hardening by the Cu particles. Due to the intricate evolution of the microstructure, aging treatments above 490 °C led to a loss in yield strength and ductility. A considerable rise in strength and a decrease in ductility were brought about by the increase in the fraction of precipitation-hardened martensitic matrix in aging treatments over 640 °C. The impact of heat-treatment pathways on aging effectiveness and tensile anisotropy was then examined. Direct aging at 482 °C for an hour had hardly any effect on wrought 17-4PH, but it increased the yield strength of AM counterparts from 436–457 to 588–604 MPa. A solid-solution treatment at 1038 °C for one hour resulted in a significant drop in the austenite fraction, which led to an increase in the yield (from 436–457 to 841–919 MPa) and tensile strengths (from 1106–1127 to 1254–1256 MPa) with a sacrifice in ductility. Improved strength and ductility were realized by a solid-solution followed by an aging treatment, achieving 1371–1399 MPa. The tensile behaviors of AM 17-4PH were isotropic both parallel and perpendicular to the building direction.

## 1. Introduction

Additive manufacturing (AM) technology for nuclear power components has been evaluated as a possible fabrication method to replace the current industrial system because it has advantages for reverse engineering and it permits domestic self-supply in the era of technology protectionism. Even with intricate structures, the AM method can create components in a net shape, making it ideal for hard-to-deform materials. Numerous researchers have attempted to use AM technology on components such as control valves in nuclear power systems on the basis of the aforementioned advantages [1].

Control valves are closely related to safety since they are essential for the reliable operation of nuclear power systems. Therefore, the functionality of the components produced using a novel AM technique should be guaranteed to ensure stable operation. The body, bonnet, cage, and plug are used to assemble the control valve. Because they operate in harsh situations with continuous friction, high pressure, and high temperatures, the body and bonnet are made of 316 L stainless steel, and the cage and plug that regulate pressure and fluid flow are made of 17-4PH stainless steel. This stainless steel is chosen because, when given the proper heat-treatment, it offers excellent mechanical performance [2]. While 17-4PH stainless steel is accordingly appropriate for use in AM technology for nuclear power components, evaluations and optimization via post processing of the mechanical properties of the parts created by this new method are insufficient.

Numerous researchers have conducted studies on manufacturing 17-4PH stainless steels using AM and their resulting properties. A. Kudzal et al. observed the effect of scanning strategies in powder bed fusion on the microstructure and tensile properties of 17-4PH steel [3]. They reported that scanning patterns changed the fraction of constituent phases and morphological characteristics, which drove the different tensile and fracture behaviors. H.K. Rafi et al. emphasized that the mechanical properties of AM 17-4PH depended on the retained austenite, which varied by the AM conditions [4]. It is well known that aging treatment is a vital post-process for PH steels because it imparts considerable enhancement [5]. Z. Wang et al. analyzed Cu particles precipitated by an aging treatment in 17-4PH using atom probe tomography [6]. They argued that the number density of Cu particles generating precipitation-hardening increased in a temperature range from 350 °C to 450 °C while it decreased at a higher temperature. S. Cheruvather et al. studied the effects of post-processing via a comparison between wrought and AM 17-4PH stainless steels [7]. The results of his study indicated that the heat-treatment for wrought steels used in industries was ineffective in AM steels, and solid-solution treatment should be optimized. We already noticed that the aging temperature profoundly influences the precipitation behaviors in 17-4PH steels, which should correlate to strengthening ability. However, studies on its application to industries are still insufficient. Since the austenite transformation temperature (above A_s_) of 17-4PH stainless steel containing austenite-stabilizing elements overlaps with precipitation temperatures [8], austenite reversion and precipitation at this overlapped temperature range should change aging efficiency [2,9] and transformation-induced plasticity (TRIP) [10] behavior, which would lead to changes in the mechanical properties. Moreover, in contrast with 17-4PH steel manufactured by conventional methods, AM 17-4PH steels reveal comprehensively different microstructures in terms of AM conditions [3,11,12]. Therefore, it is important to suggest optimized aging conditions.

Using direct energy deposition (DED), we manufactured 17-4PH stainless steels utilized in nuclear power plants for this investigation. We then aged the steels at a variety of aging temperatures ranging from 400 °C to 700 °C. To select the ideal aging temperature, hardness and tensile tests were carried out. Scanning transmission electron microscopy (STEM) and small angle neutron scattering (SANS) were used to qualitatively and quantitatively evaluate particles precipitated by selected aging treatment. Subsequently, we separated the conventional heat-treatment approach into three independent processes: direct aging, solid-solution, and solid-solution followed by aging, and we compared their effects on the AM samples with the effects on conventionally manufactured counterparts. In the fields of industry and science, this work should aid in the development of heat-treatment strategies and the optimization of aging treatments.

## 2. Materials and Methods

### 2.1. Additive Manufacturing and Heat-Treatments

**Figure 1:**
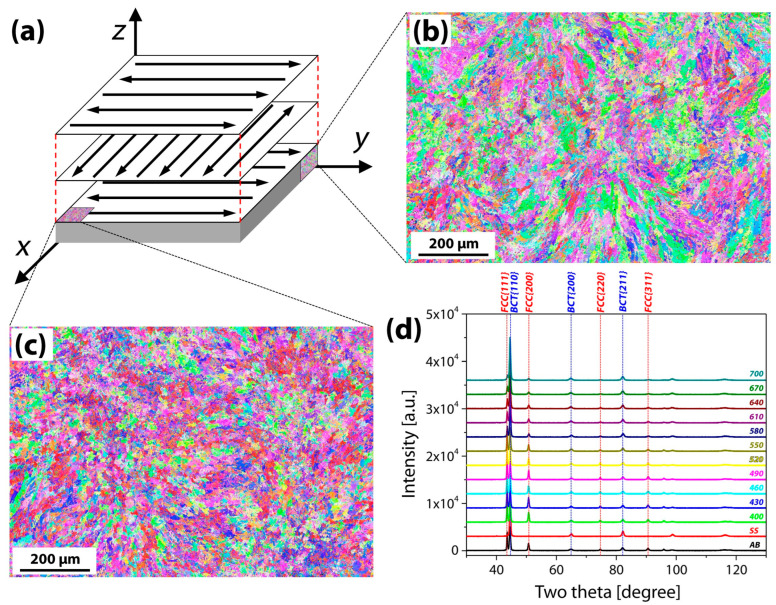
(**a**) Zigzag scanning strategy used in additively manufactured 17-4PH stainless steel, inverse pole figure maps for (**b**) cross-section, (**c**) deposited surface of as-built block, and (**d**) XRD spectrum for as-built, solid-solutionized and aged samples.

Table 1 provides the chemical composition of the 17-4PH metallic powder used for the AM in this work. Under the AM conditions listed in Table 2, it was additively produced into a 120 mm × 60 mm × 15 mm block using a zigzag building strategy (Figure 1a). Aging temperature ranges between 400 °C and 700 °C were chosen, with a 30 °C gap between each temperature, in order to evaluate the effect of temperature. The as-built (AB) sample was heated for 30 min to the target aging temperature, held for an hour, and then air-cooled. Note that the ASTM A564/A564M-19A H900 [13] heat-treatment served as the basis for these adjusted aging conditions. We then designed the following three heat-treatment routes combined with solid-solution treatment after selecting the most efficient aging treatment among the above aging temperature ranges: Direct aging (A) without solid-solution, solid-solution (SS), and solid-solution followed by aging (SSA). Note that the solid-solution treatment entails heating to 1038 °C for 30 min, residing for an hour, and finally water-quenching. Wrought 17-4PH steel was also examined as a counterpart to observe the impacts of AM.

### 2.2. Material Characterization

By using an inductively coupled plasma (ICP) and element analyzer (EA), the precise chemical composition of the AM 17-4PH stainless steel block was determined. X-ray diffraction (XRD) and electron backscatter diffraction (EBSD) were used to define the microstructure of the samples. Rietveld refinement using GSAS-II [14] was conducted on the XRD spectrum, which provided the lattice parameters and phase fractions of each sample. Vickers hardness test and a tensile test with a strain rate of 10^−3^/s were performed on a sample with a gauge length of 12.5 mm, a thickness of 1.2 mm, and an axis perpendicular to the building direction (in-plane) in order to reveal the effect of the aging temperature. Tensile samples with gauge lengths of 5.7 mm and thicknesses of 1.2 mm were machined for their axes to be perpendicular and parallel to the building direction, respectively, from the AM block to collect the mechanically anisotropic responses. A 10^−3^/s tensile test was then performed on these samples using three different heat-treatment pathways, and the results were compared to those of wrought 17-4PH steel. Precipitates in aged samples were qualitatively analyzed by scanning transmission electron microscopy (STEM) and were quantified by small-angle neutron scattering using the 18M-SANS instrument at the high-flux advanced neutron application reactor (HANARO) of the Korea Atomic Energy Research Institute (KAERI) [15]. The configuration of the wavelength and detector distance in SANS experiments (4.56 Å 3.2 m, 9 Å 9.2 m) was selected to collect a wide q-range of scattered neutrons. The observed I-Q curves were simulated by SASfit software (version 220619075431) [16].

## 3. Results and Discussion

### 3.1. Microstructural Evolution

The chemical composition of the AM 17-4PH stainless steel block analyzed by ICP and EA is presented in Table 3. It was similar to the chemical composition of metallic powder, but the Si element was not detected. This might be attributable to the formation of SiO_2_ during the AM process [17]. Figure 1 depicts the microstructures of the AM17-4PH block and heat-treated samples observed by EBSD and XRD. The average grain size is 5.17 ± 5.53 μm and distinct morphological traits were revealed. Due to the variation in the solidification rates in the melting pool, the cross-section of the AM block showed a heterogeneous distribution of grain sizes [18], which led to the unique morphology seen in Figure 1b. However, this morphology was not observed on the face of the deposit (Figure 1c). Figure 1d displays the XRD spectrum for samples that were as-built, solid-solutionized, and directly aged at each temperature. The other samples showed both body-centered tetragonal (BCT) and face-centered cubic (FCC) peaks; however, the SS (red line) samples exclusively showed BCT peaks.

**Figure 2:**
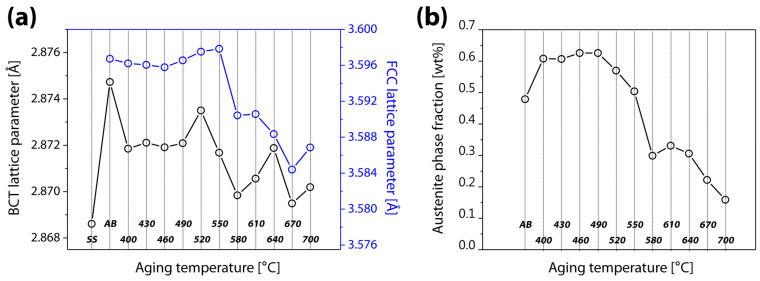
Changes in (**a**) the lattice parameters of BCT (black) and FCC (blue) and (**b**) the austenite phase fraction in terms of aging temperature.

The results of the Rietveld refinement of the XRD spectrum are illustrated in Figure 2. In comparison to sample AB, sample SS showed a substantially lower BCT lattice parameter, and it generally dropped as the aging temperature rose. The FCC lattice parameter, on the other hand, barely changed in AB up to the aging temperature of 550 °C, but it dramatically fell at higher aging temperatures. The phase fraction of retained austenite is depicted in Figure 2b. Sample AB had a notably high austenite phase fraction (47.9%). It is well known that AM conditions have a major influence on the ratio of the martensite-austenite phase fraction [3,19,20]. The phase fraction of austenite grew to 60.8% at the aging temperature of 400 °C, and this increasing tendency persisted up to the aging temperature of 490 °C (62.6%). The sample aged at 520 °C showed a relatively low fraction of retainued austenite (57%) and at increasing temperatures of aging treatment, the fraction of retained austenite continued to decline, reaching 15.9% at the aging temperature of 700 °C.

The interstitial atom concentration and residual stress are key factors in determining the lattice parameters [21,22,23,24]. Since interstitial atoms are less soluble in BCT martensite, the decrease in the BCT lattice parameter was caused by the diffusion of interstitial atoms with heterogeneous distributions into austenite with a higher solubility [2,17,25], as well as the attenuation of martensite defects (a tempering effect) [26], both of which occurred during the aging process. This effect could be applied in solid-solution treatment. The stability of austenite was strengthened by the diffusion of interstitial atoms, with increasing austenite content observed in samples aged at 400–490 °C [27]. The difference between aging temperature and room temperature (a driving force for martensitic transformation) surpassed the certain critical temperature, acting like a threshold effect, above the aging temperature of 580 °C, which led to a large rise in martensitic transformation [8]. The reduction in the FCC lattice parameter might be a result of compressive internal stress imposed on austenite by the quantitative rise in martensite with lower density [28].

### 3.2. Evolution of Tensile Behavior

**Figure 3:**
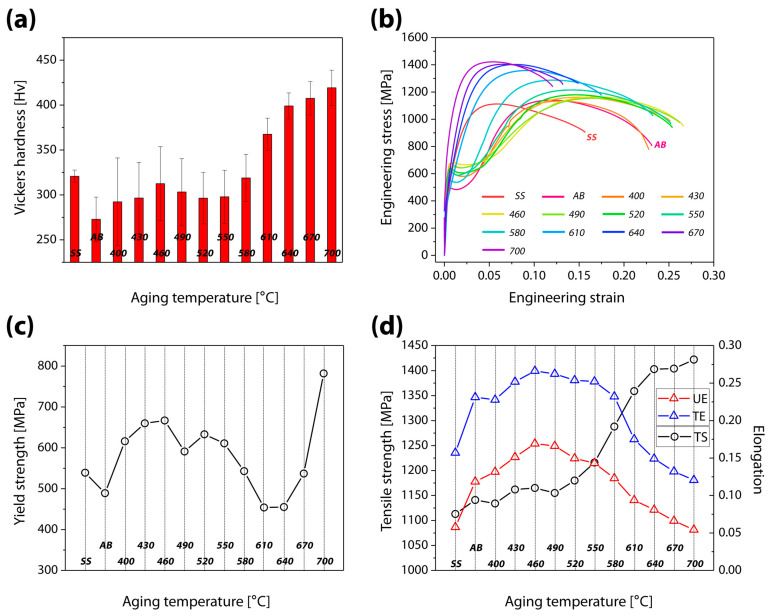
(**a**) Vickers hardness, (**b**) stress-strain curves, (**c**) yielding strength, and (**d**) tensile strength (black), uniform elongation (red), and total elongation (blue) of as-built, solid-solutionized, and aged samples.

Figure 3a shows the Vickers hardness data for the AB, SS, and aged samples. Sample AB exhibited a hardness of 273 Hv, while sample SS revealed a value of 321 Hv. These values are attributed to the elimination of soft austenite. While the sample aged at 490 °C showed decreased hardness, the aging treatment from 400 °C to 460 °C enhanced the hardness up to 313 Hv. The sample aged at 520 °C had a hardness of 296 Hv, whereas the sample aged at 700 °C had a higher hardness of 419 Hv.

Figure 3b–d display the results of the tensile test. Note that a 0.2% offset was used to determine the yield strength (YS). Sample AB yielded at 489 MPa and then showed a plateau and a low work-hardening with further strain. It had a tensile strength (TS) of 1141 MPa at a strain of 11.85% (uniform elongation, UE) and was fractured at a strain of 23.12% (total elongation, TE). The plateau after yielding in sample AB resulted from a TRIP of a sizable fraction of retained austenite because TRIP imposed a large plastic flow [10,27]. However, solid-solution treatment eliminated this effect. Although sample SS revealed a higher YS of 539 MPa than that of sample AB without a plateau, it exhibited degraded performance, showing a TE of 1113 MPa at a UE of 5.78%, and fracture at 15.7%. This indicated that sample AB with a large amount of retained austenite had an advantage in plastic deformation in comparison with sample SS, which is composed entirely of martensitic phase.

The samples were divided into two groups based on their tensile characteristics. While sample SS and the samples aged at 610–700 °C demonstrated tensile behaviors without a plateau, sample AB and the samples aged at 400–580 °C showed a clear plateau after yielding. In the group showing the plateau, the 400 °C aging treatment improved all parameters, including the YS, the TS (1175 MPa), the UE (16.92%), and the highest TE (26.63%). In the group without the plateau, the UE and the TE dropped as the aging temperature increased, with the sample aged at 700 °C showing a TS of 1422 MPa at a UE of 5.43%, and a TE of 12.06%. On the other hand, the YS decreased until the aging temperature of 610 °C (YS: 454 MPa) but increased at higher aging temperatures, reaching a YS of 782 MPa at 700 °C.

**Figure 4:**
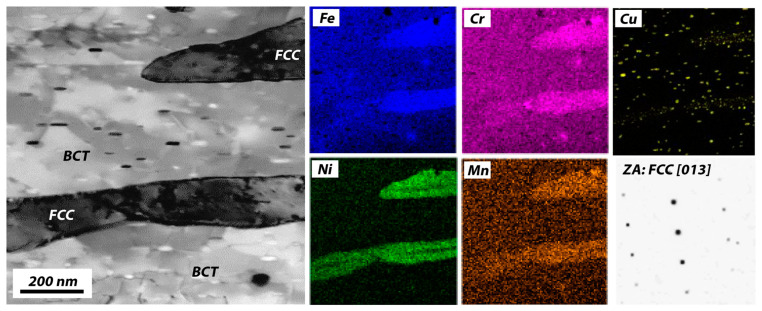
A scanning transmission electron microscopy image with energy dispersive spectroscopy maps and selected area electron pattern on the FCC phase of thes aged 17-4 PH sample.

It is widely known that precipitation strengthening by Cu particles yields improved tensile behavior [2,6,19]. In the samples that underwent the modified H900 treatment (direct aging treatment of 482 °C for an hour without solid-solution treatment), electron dispersive spectroscopy (EDS) maps of martensitic and austenitic phases and selected area electron diffraction (SAED) patterns on austenite were obtained by STEM (Figure 4). The dark area in the STEM image corresponds to FCC austenite containing strong austenite-stabilizing elements, such as Ni and Mn. Cu agglomerates induced by the aging treatment were also seen, and they were uniformly distributed regardless of the constituent phase. This contradicts an earlier study that claimed that only the martensitic phase bears Cu precipitates [29].

**Figure 5:**
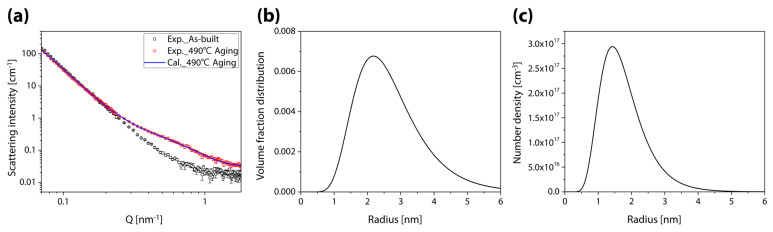
(**a**) I−Q curves of as-built and 490 °C aged samples (symbol: experimental results, line: calculated results), (**b**) volume fraction distribution, and (**c**) number density of Cu precipitates in the 490 °C aged samples.

Figure 5 illustrates SANS results that provide quantitative data on Cu precipitates. Black and red symbols, respectively, represent the scattering responses of sample AB and the sample aged at 490 °C. A clear difference in the scattering intensity between both curves can be seen within a particular q-range, indicating that particles of a particular size with a scattering contrast were formed after the aging treatment and that they correspond to Cu precipitates agglomerated during the aging treatment. We used the model in the SASfit software to quantify the Cu particles. It is assumed that Cu precipitates are spherical and log-normally distributed. The blue line in Figure 5a represents the simulation results, which accurately captured the experimental responses (red symbols). These results offered volume fraction and particle number density in terms of the radius (Figure 5b,c). The Cu particles were distributed in the samples with a radius of 0.5–5 nm, showing an average radius of 1.765 nm and a proportion of 1.492%.

At the aging temperature of 400–460 °C, an improvement in the YS, the TS, the UE, and the TE was accomplished without a trade-off between strength and ductility. The austenite and martensitic matrix were strengthened by the precipitated Cu particles after aging treatment, which also increased the YS and produced an effective TRIP behavior. It appears that the TRIP effect, which was activated at a higher YS, led to an increase in both strength and ductility [27]. Aging treatments at a temperature higher than 490 °C decreased the UE and the TE until the aging temperature of 610 °C, which is assumed to be the results of numerous and complicated effects. This temperature range encourages precipitation and a rise in the martensitic phase fraction, inducing considerable strengthening. As a result, a decrease in ductility was noted. Additionally, a drop in the YS may be caused by the recovery effect in the martensitic matrix [26]. However, the rise in the percentage of the hard martensitic matrix, the interaction of Cu precipitates with dislocations, and the TRIP effect under increased strain resulted in a significant improvement in the TS at the expense of a small amount of ductility. With a consistent decline in ductility, aging treatments from 640 °C to 700 °C clearly increased the YS and the TS. This was explained by an increase in stiffness brought on by a significant decrease in the soft austenite content, which showed an obvious trade-off between strength and ductility. Increases in TS are attributed to precipitation strengthening, whereas the reduction in ductility should be postponed by the TRIP effect of the retained austenite.

### 3.3. Solid-Solution and Heat-Treatment Path

A guide to choosing the appropriate aging temperature in terms of goals and target properties is offered by the investigation of aging treatments in a range of 400–700 °C in the above sections. It was discovered that the aging treatment at 460–490 °C successfully increased both strength and ductility. The H900 heat-treatment of ASTM A564/A564M-10a (solid-solution treatment at 1038 °C for an hour followed by aging treatment at 482 °C for an hour) is comparable to this aging temperature. Among many heat-treatment settings, the H900 heat-treatment is ideal for strengthening PH steel. We chose the H900 heat-treatment and decomposed it into each process (direct aging without solid-solution, only solid-solution, solid-solution followed by aging). This treatment was then applied to the AM 17-4PH sample and a conventional wrought 17-4PH sample (commercial alloy, CA) as a reference. Tensile anisotropy with regard to building direction was also observed. Table 4 provides an overview of the prepared samples and their tensile test outcomes.

**Figure 6:**
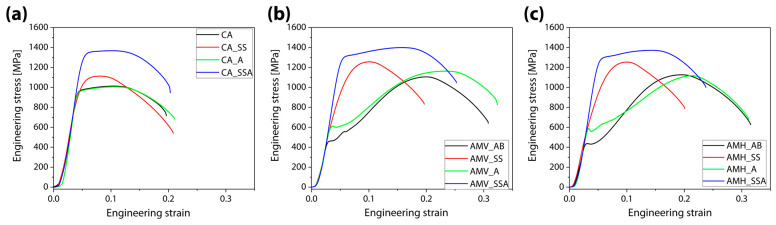
Stress–strain curves of (**a**) wrought steels, and (**b**,**c**) additively manufactured steels in terms of loading direction (parallel to the building direction: (**b**), perpendicular to the building direction: (**c**)) and heat-treatment paths (black: as-built or none, red: solid-solution, green: direct-aging, blue: solid-solution followed by aging).

**Figure 7:**
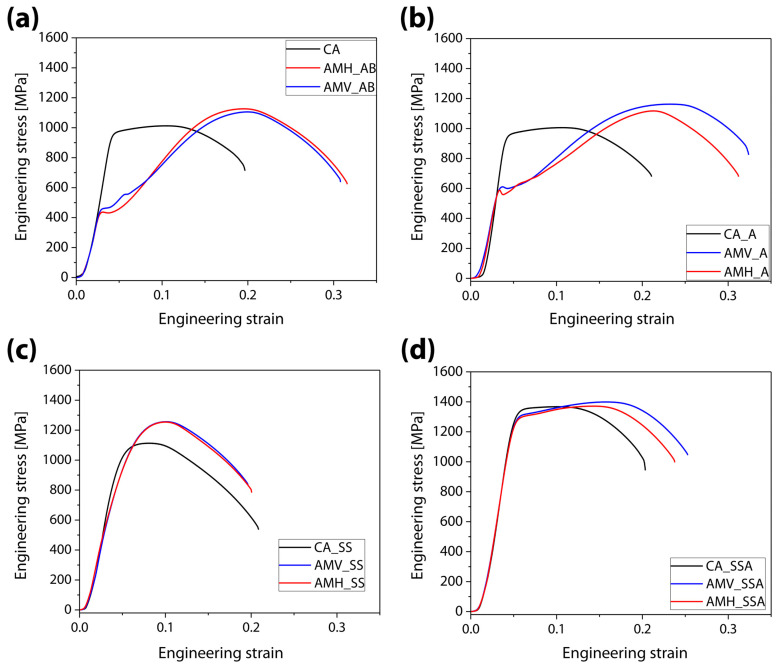
Stress–strain curves in terms of heat treatments. (**a**) as-built or none, (**b**) direct aged, (**c**) solid-solutionized, and (**d**) solid-solutionized followed by aged samples (black: wrought samples, blue: additively manufactured samples loaded along the building direction, red: additively manufactured samples loaded along an in-plane direction).

Figure 6 and Figure 7 depict the obtained tensile curves. The results of heat-treatment pathways are shown in Figure 6. The tensile behavior of wrought steel was negligibly affected by direct aging treatment. While solid-solution treatment boosted the TS, which had a value of 1113 MPa (CA_SS), it decreased the YS of CA from 958 MPa (CA) to 893 MPa (CA_SS). The aging treatment improved CA_SS, resulting in a YS of 1243 MPa and a TS of 1367 MPa (CA_SSA). The TE of all CA samples revealed a small amount of deviation. In comparison to CA samples, AM samples had a markedly different tensile behavior. Tensile curves evaluated with parallel (vertical case) and perpendicular (horizontal case) loading direction to building direction are shown in Figure 6b and Figure 6c, respectively. Direct aging treatment significantly raised the YS of the AB samples from 457 MPa to 604 MPa (AMV_A) and from 436 MPa to 588 MPa (AMH_A), but only marginally increased the TS. This showed that direct aging treatment for precipitation strengthening was ineffective for AB samples. In contrast, for both AB samples (AMV_AB, AMH_AB), solid-solution treatment considerably altered the trends of the stress–strain curves. However, it significantly lowered the UE and the TE while significantly increasing the YS (919 MPa for AMV_SS, 841 MPa for AMH_SS) and the TS (1256 MPa for AMV_SS, 1254 MPa for AMH_SS). This was attributed to the transformation from most of the retained austenite in AB samples to martensite during the aging treatment, which is consistent with a prior study [5]. With the increase of both the UE and the TE, solid-solution and aging treatments produced an effective enhancement of the YS (1234 MPa for AMV_SSA, 1215 MPa for AMH_SSA), and the TS (1399 MPa for AMV_SSA, 1371 MPa for AMH_SSA).

Figure 7 shows the impacts of loading direction on heat-treatment routes using the stress–strain curves of CA samples as counterparts. Although anisotropic tensile behaviors were hardly observed in the AM samples, these samples showed a clear distinction from the CA samples. Due to retained austenite, the AB samples exhibited a plateau and low work-hardening after yielding, but the CA samples revealed typical tensile behaviors observed in martensite steels (Figure 7a). This trend persisted in samples that were directly aged (Figure 7b). AMV_SS and AMH_SS, on the other hand, not only had a comparable tensile tendency to that of CA_SS, but also showed an improved YS, TE, and UE (Figure 7c), which are assumed to originate from microstructural improvements brought about by AM [10]. In the SS samples, the aging treatment produced the greatest improvement in tensile properties without sacrificing ductility (Figure 7d).

Significant changes in the microstructure led to variations in tensile behavior in terms of the heat-treatment pathway. As previously indicated, AB samples had a significant amount of retained austenite (Figure 1d), which led to a significant amount of plastic flow during deformation by TRIP. As a result, AB samples showed a lower YS than other samples. After being hardened by newly transformed martensite by TRIP, AB samples showed a greater TS than CA samples (Figure 7a). In AB samples, direct aging treatment resulted in negligible precipitation hardening, which may have been caused by the failure of Cu precipitates to reinforce the martensitic matrix. As a result of the transformation from soft austenite to hard martensite in AB samples by solid-solution treatment, the TS increased in SS samples (Figure 1d), whereas the UE was greatly decreased as a result of brittle martensite. The martensitic matrix’s excellent precipitation hardening gave the SSA samples their exceptional strength. The heterogeneous microstructure imposed by AM resulted in a higher TS and UE in as-built and direct-aged samples compared to those of CA. The large variety of grain sizes caused plastic flow and hardening at the same time, improving the tensile properties along with the TRIP of finely dispersed retained-austenite. However, the heterogeneous microstructure of the AM samples was eliminated by the solid-solution treatment, leaving the samples with a typical martensitic phase. As a result, those tensile behaviors hardly differ from CA [5,10].

## 4. Conclusions

This study examined the effects of effective aging temperatures and effective heat-treatment pathways on the tensile properties of additively manufactured 17-4PH stainless steels. A large fraction of retained austenite (47.95%) was present in as-built samples prepared by direct energy deposition, which led to a low yield strength and a plateau after yielding. Precipitation strengthening with Cu particles produced an improvement without a trade-off between strength and ductility, although the aging treatment of 400–460 °C increased the fraction of retained austenite up to almost 60%. Due to a combination of precipitation hardening, a drop in the austenite proportion, and recovery effects in the martensitic matrix, the aging treatment at 490 °C resulted in a decrease in the YS as well as elongation. The percentage of martensitic matrix that was precipitated-hardened increased at aging temperatures over 640 °C, which resulted in a noticeable decrease in ductility and an increase in the yield and tensile strengths without a plateau. Direct aging treatment at 482 °C for an hour had no effect on the tensile behavior of wrought steel, but it considerably increased the yield strength of the additively manufactured samples. In wrought and additively manufactured samples, a solid-solution treatment at 1038 °C for an hour decreased the fraction of retained austenite, which increased the yield and tensile strengths but decreased ductility. The strength and the ductility of wrought and additively manufactured samples were greatly improved by the solid-solution and aging process. The tensile behaviors of additively manufactured samples were negligibly affected by the loading direction in relation to the building direction.

## Figures and Tables

**Table 1:** Chemical composition of 17-4PH metallic pow der.

Fe	Cr	Ni	Cu	Mn	Si	Nb	Mo	C	N	S	P
Bal.	16.8	4.63	4.66	0.72	0.6	0.25	0.1	0.05	0.1	0.005	0.01

**Table 2:** Manufacturing conditions of direct energy deposition for 17-4PH samples.

Substrate	SM45C	Beam Power	425 W
Beam width	800 μm	Powder feeding	3 g/min
Atmosphere	Ar	Strategy	Zigzag

**Table 3:** Analyzed chemical composition of additively manufactured 17-4PH stainless steel.

Fe	Cr	Ni	Cu	Mn	C	N	O
73.062	16.123	4.910	5.013	0.728	0.041	0.070	0.053

**Table 4:** Tensile properties of AM and wrought 17-4PH stainless steel in terms of heat-treatment paths and loading direction.

Manufacturing	Loading Direction	Heat Treatment	Abbreviation	0.2% Offset Yield Strength (MPa)	Tensile Strength (MPa)	Uniform Elongation	Total Elongation
Wrought	Extrusion	None	CA	958	1012	0.111	0.197
	Extrusion	Direct aging	CA_A	914	1006	0.108	0.211
	Extrusion	Solid-solution	CA_SS	893	1113	0.082	0.209
	Extrusion	Solid-solution & aging	CA_SSA	1243	1367	0.106	0.203
AM	Parallel to building direction (Vertical)	None (As-built)	AMV_AB	457	1106	0.2	0.308
		Direct aging	AMV_A	604	1162	0.24	0.324
		Solid-solution	AMV_SS	919	1256	0.103	0.196
		Solid-solution & aging	AMV_SSA	1234	1399	0.162	0.253
	Perpendicular to building direction (Horizontal)	None (As-built)	AMH_AB	436	1127	0.196	0.316
		Direct aging	AMH_A	588	1117	0.215	0.312
		Solid-solution	AMH_SS	841	1254	0.099	0.201
		Solid-solution & aging	AMH_SSA	1215	1371	0.148	0.238

## Data Availability

The data that support the findings of this study are available upon request.
